# Depth Echolocation Learnt by Novice Sighted People

**DOI:** 10.1371/journal.pone.0156654

**Published:** 2016-06-03

**Authors:** Alessia Tonelli, Luca Brayda, Monica Gori

**Affiliations:** 1 U-VIP: Unit for Visually Impaired People, Science and Technology for Children and Adults, Istituto Italiano di Tecnologia, Via Morego 30, Genova, Italy; 2 Robotics, Brain and Cognitive Sciences Department, Fondazione Istituto Italiano di Tecnologia, Via Morego 30, 16163, Genova, Italy; Kyoto University, JAPAN

## Abstract

Some blind people have developed a unique technique, called echolocation, to orient themselves in unknown environments. More specifically, by self-generating a clicking noise with the tongue, echolocators gain knowledge about the external environment by perceiving more detailed object features. It is not clear to date whether sighted individuals can also develop such an extremely useful technique. To investigate this, here we test the ability of novice sighted participants to perform a depth echolocation task. Moreover, in order to evaluate whether the type of room (anechoic or reverberant) and the type of clicking sound (with the tongue or with the hands) influences the learning of this technique, we divided the entire sample into four groups. Half of the participants produced the clicking sound with their tongue, the other half with their hands. Half of the participants performed the task in an anechoic chamber, the other half in a reverberant room. Subjects stood in front of five bars, each of a different size, and at five different distances from the subject. The dimension of the bars ensured a constant subtended angle for the five distances considered. The task was to identify the correct distance of the bar. We found that, even by the second session, the participants were able to judge the correct depth of the bar at a rate greater than chance. Improvements in both precision and accuracy were observed in all experimental sessions. More interestingly, we found significantly better performance in the reverberant room than in the anechoic chamber. The type of clicking did not modulate our results. This suggests that the echolocation technique can also be learned by sighted individuals and that room reverberation can influence this learning process. More generally, this study shows that total loss of sight is not a prerequisite for echolocation skills this suggests important potential implications on rehabilitation settings for persons with residual vision.

## Introduction

Echolocation can be compared to a biological sonar used by several kinds of animals: the species most known to adopt this peculiar technique are dolphins and bats. Echolocating animals use self-generated sounds to measure the time delay between their own sound emission and any echo reflected by the environment. The relative intensity of the sound received by each ear as well as the delay in the time it takes for the sound to arrive to the two ears provide information about the horizontal angle (azimuth) from which the reflected sound waves arrive. Similarly, spectral coloration of the reflecting object can provide us with information regarding its nature. With this technique animals are able to live in environments where the visual channel cannot be used as a primary source of spatial information. This permits them to carry out essential activities such as hunting or navigating even in complete darkness.

Surprisingly, a few blind humans have learned to use echolocation to replace vision (for a review [1]). This allows them to perceive aspects of the surrounding environment that they could not perceive otherwise.

Several studies have demonstrated that both sighted and blind people are able to process object features such as distance or position [2–6], motion [7, 8], size [4, 9, 10], shape [8, 11] and kind of material [12, 13] by using self-generated sounds (mouth-clicks or finger-snapping) or by listening to pre-recorded clicks and echoes in virtual environments.

A recent study [14] compared the performance of expert echolocators and blind and sighted people with no previous experience of echolocation, in a space bisection task. It was found that, contrary to non-expert blind echolocators, expert blind echolocators performed the spatial task with similar or even better precision and accuracy than the sighted group. This supports the hypothesis that echolocation recalibrates and even refines the ability of blind individuals to represent sounds in complex spatial configurations and compensates for their lack of vision.

Several studies of neuroimaging have revealed that expert echolocators recruit the same visual areas usually activated by visual stimuli in sighted people [8, 15, 16]. Furthermore, echolocation-specific activity has been reported in the calcarine cortex, a brain region typically used by vision [8].

More importantly, this technique seems to provide benefits in the daily activities of echolocators, fostering their social inclusion. For example, it has been shown that echolocation is associated with higher salaries and mobility in unfamiliar places, which play a role in the successful adaptation to vision loss [17].

To date it is not clear whether sighted individuals can learn this technique in a similar way. Most of the studies on echolocation that have involved sighted subjects have compared their performances with those of echolocators and blind non echolocators, without training them. On the other hand, there are a number of works that have attempted to address this question, by studying the echolocation learning process of sighted people. Teng and Whitney (2011), for example, found that sighted novices improved their echolocation ability with regard to size discrimination across multiple sessions, but they also found that there was great variability across participants. Moreover, in an echolocation task designed to assess spatial acuity, the performance of participants ranged from complete inability to performance approaching the level of experienced blind echolocators [4]. Thaler et al. [9] confirmed the results obtained by Teng and Whitney (2011) in the size discrimination task and also found a positive correlation between the ability to perform a size discrimination task with echolocation and the vividness of visual imagery in sighted people.

Echolocation is fundamental for navigation mainly because object distance can be inferred to avoid collision. For this reason, some studies have investigated the learning process behind echolocation learning of objects’ distances [18–20], in a virtual echo-acoustic space using pre-recorded noise and echoes produced by a reflective surface. Schenkman and Nilsson [21], in their first experiment, investigated how some acoustic information (loudness and pitch) could influence the ability to detect an object at different distances (100, 200 and 300 cm) in a two-alternative forced-choice discrimination task. They found that as long as the pitch component was present, listeners were able to perform the task, even if there was a strong effect of distance to object, i.e. the performance of subjects decreased with increasing distance, highlighting the importance of repetition of pitch for close distances, less than 2 m. These finding were confirmed by Rowan et al. [6]. In an identifying right-versus-left lateral position task, using pre-recorded bands of noise, they found that accuracy in judgment decreased with increasing distance, and from distances of 2 m or more, the participants’ performance was random. The lateral position of the surface was easily identified in the "angled" condition where the flat face of the surface was positioned so that reflected sounds were directed towards the participant, so reflection paths arrived specular to both ears. Performance was lower in the ‘flat’ condition, in which the surface's flat face produced different paths to the two ears of the participant, causing more complex binaural cues [22]. Moreover, they suggested that performance was due to high-frequency cues and longer auditory signals (400 ms), that improve performance compared to short signals (10 ms), at least for a distance below 1 m.

If the ability to echolocate is the result of training (and not a combination of visual deprivation and echolocation training), we may also expect to find an improvement of echolocation skills in sighted individuals after training. To test this theory, we investigated the ability of naive sighted people to discriminate objects less than 2 m away, using self-generated clicks to echolocate. The stimulus was inspired by previous works showing that sighted people are good at detecting an object within 2 m in a discrimination task [5, 6, 21]. We used a depth-echolocation task in which the participants had to judge the depth of five bars of poly-methyl methacrylate (PMMA) placed in front of them in five different positions (closest 30 cm, furthest 150 cm).

As we have seen above, previous works have demonstrated that under specific circumstances sighted individuals can also develop some echolocation skills. However, to date it is not clear whether specific features (such as features of the room and of the click) can influence the quality and the velocity of learning. To address this matter, here we also studied the influences of room reverberation and type of click on the learning process.

Regarding reverberation, in 2010 Schenkman and Nilsson [5] studied echolocation by using sounds recorded in a reverberant room compared with those recorded in an anechoic chamber. They found that the proportion of correct responses of blind echolocators was slightly better for the sounds recorded in a reverberant room. The results support the general idea proposed by Gibson [23], known as perceptual theory, the idea that a surplus of information makes perceptual tasks easier to perform for the subject, while a lack of information makes perception ambiguous and more difficult. In a reverberant room the sound generated by echolocation is not only reflected by the surface of the stimulus, but also by the walls, ceiling and floor of the room creating reverberation. This surplus of information could help to perform the task, as suggested by Gibson [23], by adding information to the echoes produced by stimuli, but at the same time it could "interfere" by adding noise, therefore making the task more difficult, similar to the way that background noise can make it difficult to separate sounds based on their location [24]. So the results obtained by Schenkman and Nilsson [5] could be due to a short reverberation time (T_60_ = 0.4s), and it is possible that in rooms with longer reverberation times performance would be impaired rather than improved [1]. On the other hand, in an anechoic chamber the echo is produced only by the surface of the object of interest: the sound that is not reflected by the stimulus is absorbed by the walls, thus, in principle, making the task easier, but at the same time the perceptual theory would not be supported. Despite this, expert echolocators, who independently developed this technique, are able to echolocate in environments that are far from being anechoic. In summary, it is not clear which environmental cues can contribute to echolocation learning, especially in sighted individuals. To investigate this, here we carried out a depth echolocation task in both an anechoic chamber and a reverberant room.

Regarding the influence of the type of click on the echolocation learning process Rojas et al. [25, 26] studied different types of echolocation signals, such as mouth clicks, clapping of hands etc. They analyzed the physical properties of the signals and administered questionnaires to naïve sighted people asking, for example, the objective difficulties of hearing sound variations in front of an obstacle and the subjective appreciations of the quality of the sounds, using a scale from 1 to 5. Contrary to what was found previously [5, 6], they suggest that palatal clicks are “*almost ideal signals*” for human echolocation, thanks to the combination of a relatively simple waveform and rich spectral content, allowing, for example, accurate distance estimation [26]. On the other hand, hand claps and finger snaps, when properly produced, can be a low-accuracy alternative for detecting distant obstacles, but they prevent hands from performing other tasks [25]. The prevalence of one clicking mode over another has not yet established in the literature. To better investigate this point in this work the subjects were asked to choose between two ways of producing the sound signal to perform the depth task: using the mouth (palatal mouth clicks) or by using the hands (finger snap or knuckle vacuum pulse).

We therefore checked for a possible superiority of mouth click over finger snap.

## Materials and Methods

### Participants

A total of 18 sighted participants (9 females and 9 males, with an average age of 29.9, sd = 0.95) were recruited to participate in our experiment. We verified that participants exhibited no hearing impairment with a pre-test session. In this session, we played tones through a pair of Philips SHL3000PP headphones at right and left ears separately; the tones, randomly played, were between 200 Hz and 16 kHz, with an intensity between 10 and 13 dB HL. The tones were played using the software EarTest1.0 running on a standard DELL PC. The intensity levels were preliminarily calibrated by playing the tones through the headphones while recording them with the microphone of a calibrated Delta Ohm sound level meter (HD2010UC/A). The recorded intensity levels were converted from dBA to dBHL according to standard conversion factors. Specifically, to obtain a realistic calibrated sound intensity, we simulated the volume which couples the headphone speaker with the ear by properly surrounding the microphone of the level meter by a cup made with synthetic rubber, which was put in close contact with the headphones. We verified that participants were able to hear the played tones. Participants have no cognitive impairment. All participants gave written informed consent before starting the test. The study was approved by the ethics committee of the local health service (Comitato etico, ASL 3, Genova). The participants received instructions on how to produce the echolocation signals, either by tongue click or finger snap. They were free to choose the technique they preferred. The echolocation sound was naturally produced, using no external device.

### Stimuli

**Fig 1:**
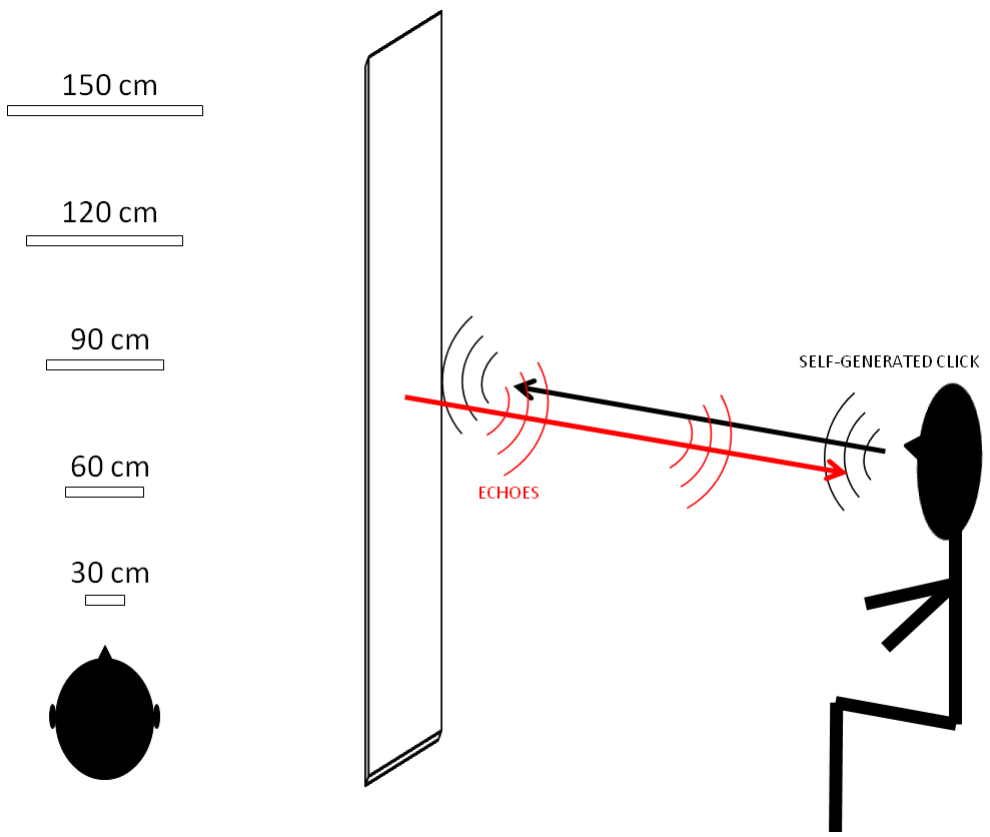
Set-up design. On the left, the five positions of the five different bars are illustrated. The first position was at 30 cm (bar size: 40cm x 6 cm) in front of the participant. The second position was at 60 cm (bar size: 72 cm x 11 cm). The third position was at 90 cm (bar size: 108 cm x 16 cm). The fourth position was at 120 cm (bar size: 145 cm x 22 cm). The fifth position was at 150 cm (bar size: 180 cm x 27 cm). On the right, there is the path of the self-generated click (black arrow) reflected by the bar producing way back echoes (red arrow).

Five rectangular bars were presented to all participants with the longer side placed vertically (Fig 1A). One of the five bars was chosen randomly and positioned at one of five possible depths: the first position was at 30 cm (bar size: 40cm x 6 cm) in front of the participant; the second was at 60 cm (bar size: 72 cm x 11 cm); the third was at 90 cm (bar size: 108 cm x 16 cm); the fourth at 120 cm (bar size: 145 cm x 22 cm) and the fifth position was at 150 cm (bar size: 180 cm x 27 cm). All bars had the same thickness (0.5 cm). When we observe an object the size of the retinal image varies with object distance. We decided to keep the auditory angle of the bars in the five possible depths constant because a previous study [27] demonstrated that expert echolocators exhibit this same phenomenon in identifying the magnitude of an object through echolocation. We decided to increase the bar size with increasing depth to ensure that the main variable possibly affecting judgments would be distance. When keeping the dimensions of a target reflecting object untouched, while distance was varied, it was shown that recognition rate drops significantly [10]. Rice et al. measured performance of a detection task, i.e. subjects had to report whether a reflecting target was in front of them or not. Moreover, keeping size constant removes a potential confounding factor in depth judgments, as speculated by Teng & Whitney [4]: subtended angle is a salient metric in size discrimination tasks across distances. When analyzing the results of Rice et al. [10], it can be noted that psychometric curves indicating echolocation performance as a function of both distance and target size have something in common: points at equal recognition rate have very similar acoustical angles. However, only target detection skills only, while not distance estimation capabilities, were considered in the study of Rice et al. [10]. We can therefore assume that reflecting objects which respect the same subtended angle are in principle similarly detectable: therefore distance judgments are minimally influenced by target “visibility”, rather by all that is left, that is the contribution of echoes.

Therefore, our bars subtended a constant acoustical angle of around 10 degrees in azimuth and of 62 degrees in elevation. A wooden structure held the bars vertically. Some magnets allowed for a quick change of the bars during the trails. Each bar was made of PMMA, which has good characteristics of reflection of sound waves.

Half of the participants performed the task in an anechoic chamber (4.8 m x 3.2 m x 2.73 m) and the other half in a reverberant room (4.6 m x 6 m x 4 m). The floor of the reverberant room was covered by parquet, and in turn completely covered by a 5mm polyester carpet; the walls were concrete, more than 50cm thick and plastered. The room had three exits: three doors in solid wood, and one window, covered by solid wood panels. The high ceiling was flat. The T60 of the reverberant room was about 1.4 seconds.

### Procedure

In both rooms, the participants were seated on a chair placed in the middle of the first half of the room, with a wall just behind them. They were facing the center of the room. They were instructed on how to generate both echolocation sounds (mouth-click and finger-snaps); they then chose only one of the two techniques. They practiced for a few minutes to generate sounds as similar as possible to each other. Since half of the participants decided to use the mouth-click and half the finger-snaps, we compared the performances obtained by the two techniques, in order to identify possible differences.

After the practice session, participants were blindfolded and given ear-bud headphones, which played mixed music. This prevented them from hearing any acoustic cues about the targets being moved by the experimenter. Three sessions were performed in two days. The first day the participants performed two sessions. The first session was a training session, before each trial the experimenter randomly placed one of the bars, at one of the five possible depths, in front of the participants. Participants were asked to remove the headphones and were given a maximum of 20 s to scan the object using the chosen echolocation technique. They had to respond verbally, reporting the depth of the bar with integer numbers from 1 to 5, where 1 was the nearest depth and 5 the furthest. In the training sessions, participants received feedback on their response: if the response was correct the experimenter confirmed the number of the position, otherwise the experimenter gave the number associated to the correct location of the bar. Each depth was repeated randomly twelve times for a total of sixty trials. One session lasted around 90 minutes.

After a 15-minute break, the participants performed the first experimental session. Here the same task was performed, but no feedback was provided. Again, 60 trials were performed for a duration of about 90 minutes.

Two days after the first two sessions, the participants were recalled to perform the second and last experimental session, which was identical to the first session in terms of trial number and duration.

## Results

**Fig 2:**
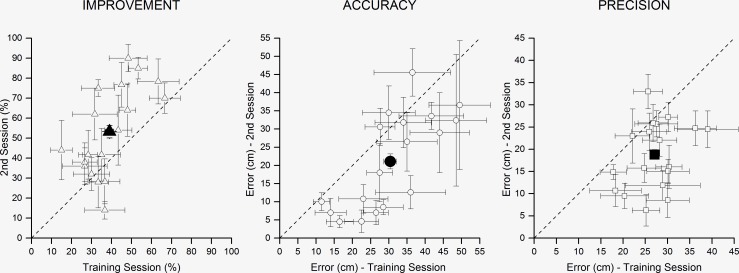
Results obtained by all the participants. (A) In the graph the percentage of correct responses obtained in the training session (x) and in the second experimental session (ordinate) are presented. (B) The scatter plot in the middle shows the accuracy with which the task was carried out in the training session (x) and in the second experimental session (ordinate). (C) The scatter plot on the right shows the precision with which the task was carried out in the training session (x) and in the second experimental session (ordinate). Filled in symbols are the average; open symbols are the performance of single subjects.

Consistent with previous studies [4–6, 9], our data show a progressive improvement in echolocating depth in sighted people after a training session. Fig 2 compares the results of the training session with the second experimental session (filled symbol is the mean; open symbols are the single performances—for the three measurement was conducted a one-way ANOVA with factor *Session* (training session X first experimental session X second experimental session). We can see that during the session is an improvement not just in the percentage of correct responses (Fig 2A) that is above the chance level (F_(3,18)_ = 6.97, p <0.01), but also in accuracy (Fig 2B) and precision (Fig 2C), which both show a significant improvement (respectively: F_(3,18)_ = 6.29, p <0.01 and F_(3,18)_ = 14.41, p <0.0001).

**Fig 3:**
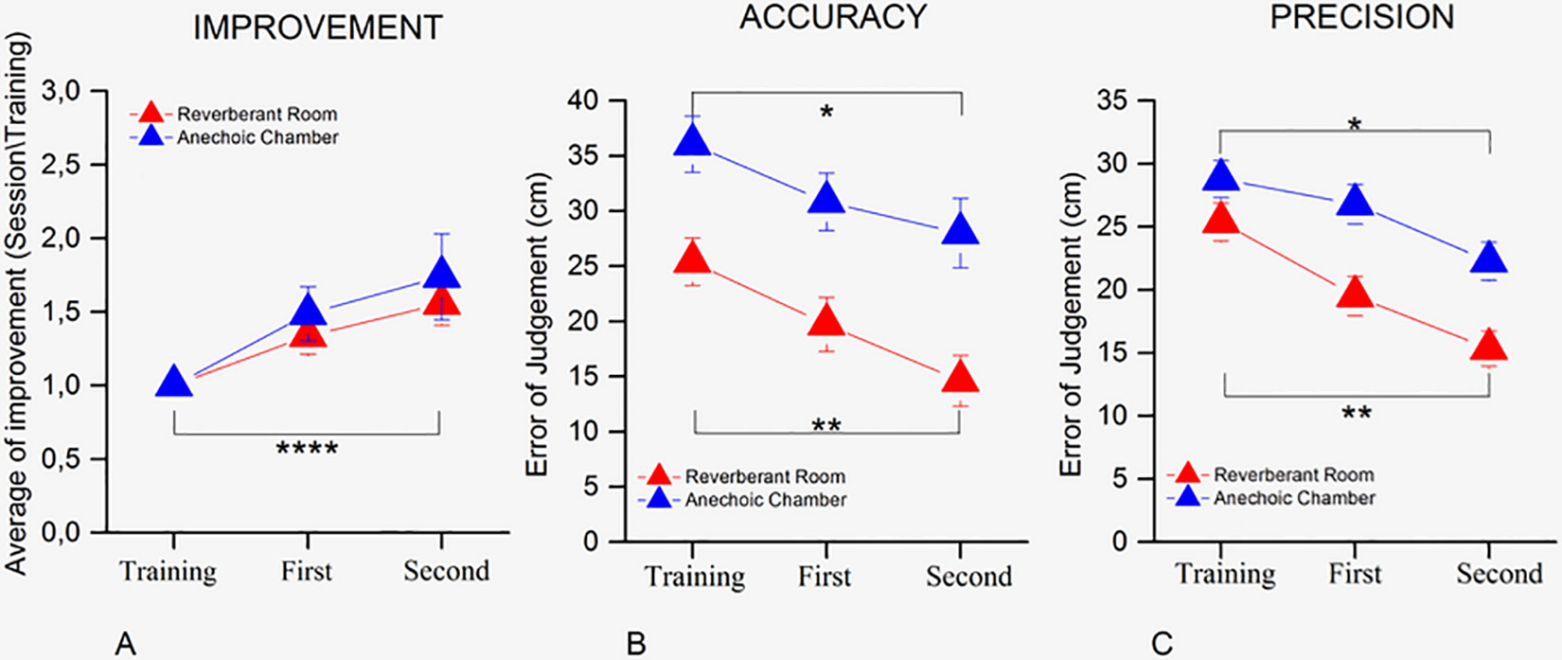
Comparison between types of rooms. (A) Shows the average ratio of improvement of the performance in both the experimental sessions. It compares the improvement for the type of room in which the participants performed the task: anechoic chamber (blue symbols) and reverberant room (red symbols). The improvement was obtained by dividing the results of all the sessions by the result of the training session. (B) The scatter plot shows the accuracy with which the task was carried out in all the sessions in the anechoic chamber (blue symbols) and in the reverberant room (red symbols). (C) The plot show the average error of judgment in precision for each rooms: anechoic chamber (in blue) and reverberant room (in red). (****)Indicates a significant difference, *p* < 0.0001. (**) Indicates a significant difference, *p* < 0.01. (*) Indicates a significant difference, *p* < 0.05.

Later, we split the sample into two groups, comparing the performance in the two rooms (Fig 3): nine participants performed the task in an anechoic chamber and nine in a reverberant room. We calculated the average ratio of improvement by dividing the percentage of correct responses of each experimental session by the percentage of correct responses of the training (Fig 3A—a mixed model two-way (3 x 2) ANOVA with within factor *Session*—training Vs 1st Session Vs 2nd Session—and between factor *Type of room*—anechoic Vs reverberant room), for the anechoic chamber (in blue) and the reverberant room (in red), and we found one main effect between the sessions (F_(4,18)_ = 17.03, p < 0.001), but not between the type of rooms (F_(4,18)_ = 0.001, p = 0.98). Concerning accuracy (Fig 3B) and precision (Fig 3C) of the error of judgment obtained by the participants in the anechoic chamber and the reverberant room (for both precision and accuracy a mixed model three-way (3 X 2 X 5) ANOVA was ran with within factor *Session* -training Vs 1st Session Vs 2nd Session—and between factor *Type of room*—anechoic Vs reverberant room—and *Position*—1st position Vs 2nd Position Vs 3rd Position Vs 4th Position Vs 5th Position) we found a significant difference for both values between sessions (accuracy, F_(6,54)_ = 15.0215, p < 0.0001; precision, F_(6,54)_ = 14.42, p < 0.0001), but in this case there was also a significant difference for the type of room in which the task was performed (accuracy, F_(6,54)_ = 6.0552, p < 0.05; precision, F_(6,54)_ = 5.87, p < 0.05), which shows that the reverberant room, is the best environment for learning echolocation.

**Fig 4:**
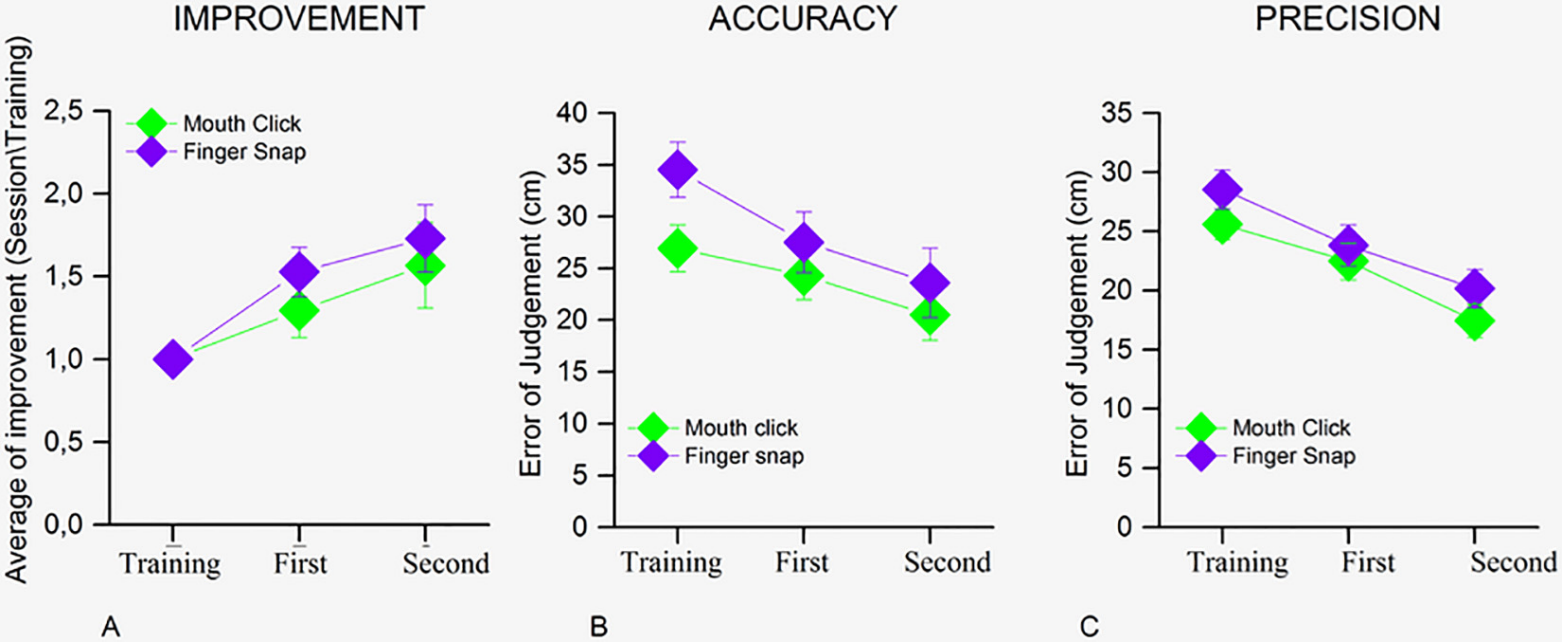
**Comparison between type of clicks** (A) Shows the average of improvement of the performance in both the experimental sessions. It compares the improvement for the type of click used by the participants in the task: mouth-click (green symbols) and finger-snap (violet symbols). The improvement was obtained by dividing the results of all the sessions by the result of the training session. A mixed model two-way (3 x 2) ANOVA with within factor *Session* (training Vs 1st Session Vs 2nd Session) and between factor *Type of room* (anechoic Vs reverberant room) was used as statistical analysis. (B) The scatter plot shows the accuracy with which the task was carried out in all the sessions using mouth-clicks (green symbols) and finger-snaps (violet symbols). (C) The bars are the average error for precision for each the five positions of the task using mouth-clicks (green symbols) and finger-snaps (violet symbols).

As well as analyzing the performance obtained in the two rooms, we investigated the results for the different types of techniques used to produce the echolocation signal. The sample was divided into two groups of nine participants each: those that decided to echolocate using mouth-click and those that used finger-snap. We calculated the average ratio of improvement by dividing the percentage of correct responses of each session by the correct responses in the training (Fig 4A—a mixed model two-way (3 x 2) ANOVA with within factor *Session*—training Vs 1st Session Vs 2nd Session—and between factor *Type of room*—anechoic Vs reverberant room) for the mouth-click (in green) and the finger-snap (in violet). As shown by the previous data, also in this case a significant effect between the sessions was found (F = 17.03, p < 0.001), where the performance in the second experimental session we significantly better compared to the training session. The type of click, however, was not shown to affect the results (F = 1.78, p = 0.19).

Conversely, accuracy (Fig 4B) and precision (Fig 4C) of the error of judgments obtained by the participants that used the finger-snap or the mouth-click to echolocate did not indicate a significant difference between the two types of technique used (respectively F_(6,54)_ = 1.06, p = 0.318 and F_(6,54)_ = 0.71, p = 0.411). However, a significant effect between sessions (accuracy, F_(6,54)_ = 15.19, p < 0.001; precision, F_(6,54)_ = 13.72, p < 0.001) was present (a mixed model three-way (3 X 2 X 5) ANOVA was ran with within factor *Session* -training Vs 1st Session Vs 2nd Session—and between factor *Type of room*—anechoic Vs reverberant room—and *Position*—1st position Vs 2nd Position Vs 3rd Position Vs 4th Position Vs 5th Position).

**Fig 5:**
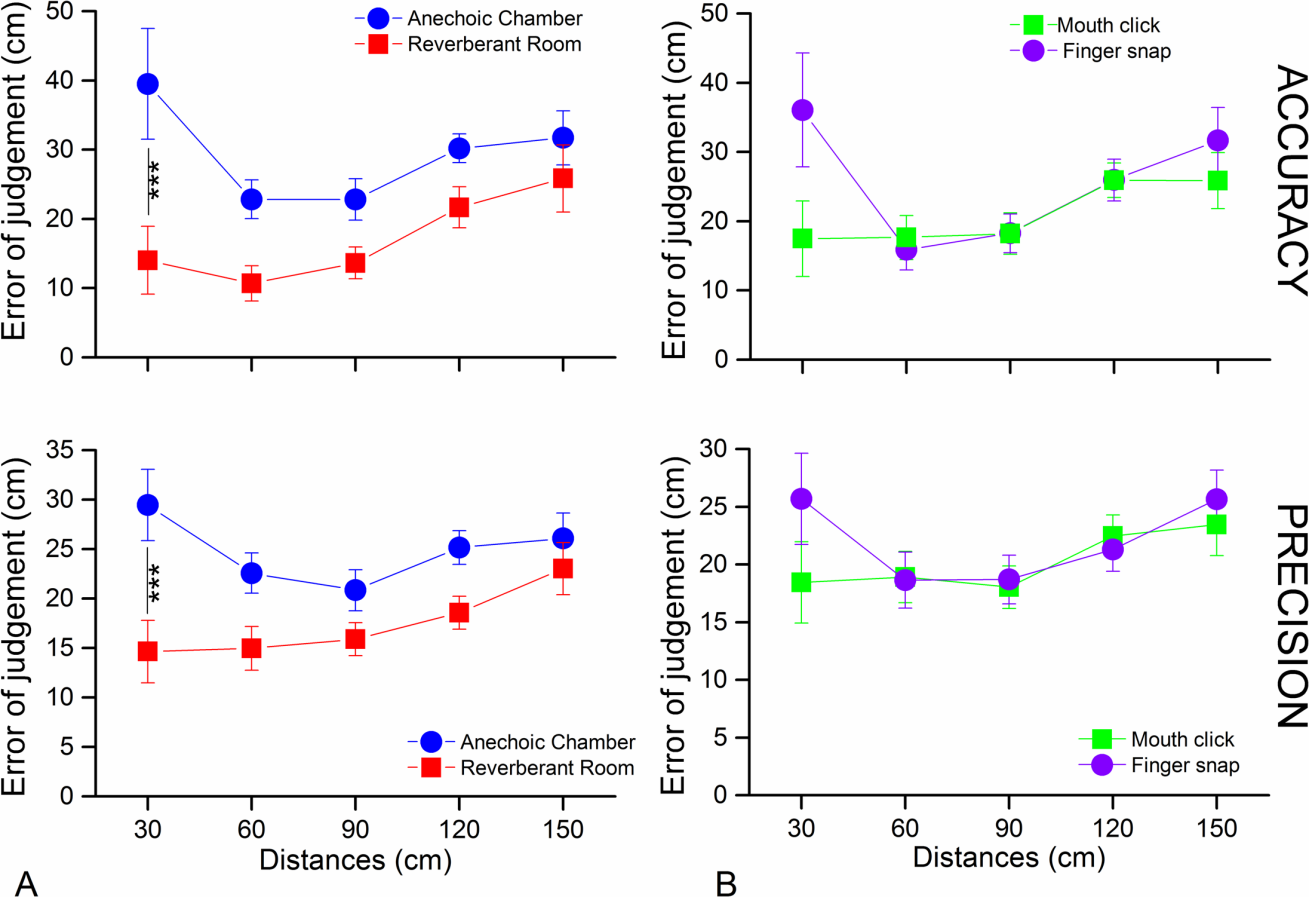
Accuracy and precision for each depth for the two experimental sessions. (A) Shows the average error for accuracy (above) and precision (below) in the anechoic chamber (in blue) and reverberant room (in red). Comparing each depth in the two rooms the only significant difference is for the depth at 30 cm. (B) The scatter plots represent the average error for accuracy (above) and precision (below) for each position divided for the type of click. (***) Indicates a significant difference (*p* < 0.001).

In Fig 5, we investigated whether there were differences between the five depths used for the two pair of groups compared (anechoic vs. reverberant room and finger snap vs. mouth click) in precision and accuracy, taking into account just the two experimental sessions. For the first group (anechoic vs. reverberant room–Fig 5A) we found a significant difference between the anechoic chamber and the reverberant room for both accuracy (F_(5,18)_ = 10.59, p = 0.003) and precision (F_(5,18)_ = 10.07, p < 0.004) and also between the different depths (accuracy F_(5,18)_ = 5.3, p < 0.01; and precision F_(5,18)_ = 3.8, p < 0.01). In particular, the data show that participants found it easier to detect the nearest position (30 cm) in the reverberant room than in the anechoic chamber (p < 0.001). For the second pair (finger snap vs. mouth click–Fig 5B), the type of click used did not significantly affect the results (accuracy F_(5,18)_ = 1.15, p = 0.29; and precision F_(5,18)_ = 0.43, p = 0.51), but there still remained a significant effect for depth (accuracy, F_(5,18)_ = 5.38, p < 0.001; and precision, F_(5,18)_ = 3.66, p < 0.01). In addition, a significant interaction between type of click and position was found for accuracy (F_(5,18)_ = 3.19, p < 0.05), but not for precision (F_(5,18)_ = 1.53, p = 0.2).

## Discussion

The aim of the current study was to investigate: i) if novice sighted people are able to learn how to echolocate in a depth task and ii) whether environmental cues, such as the room type, or sound production, i.e. the type of echolocation sound, modulate the performance of the depth echolocation task.

The first result of this study is that people who are experiencing echolocation for the first time, are able to estimate the depth of objects after just one hour of training. This result is in line with previous studies that investigated learning skills in sighted individuals by using other tasks, such as size discrimination and auditory acuity [4–6, 9]. Moreover, the improvement occurs not only in terms of percentage of correct responses, but also in terms of accuracy and precision. Thus, even sighted individuals can learn to discriminate objects relatively close to each other in depth through echolocation, with good accuracy and precision.

Secondly, room reverberation seems to influence depth perception in novice sighted echolocators: accuracy and precision were lower in the anechoic chamber than in a reverberant room. However, we found that the ratio of correct responses did not differ significantly between the two rooms.

These findings are partially consistent with the results of Schenkman and Nilsson (2010 [5]). They observed that the proportion of correct responses was slightly better in a "conference" room (reverberant room) compared with an anechoic chamber, while we did not find this difference. The different results probably stem from the fact that in their study, Schenkman and Nilsson measured distances from 50 cm to 500 cm and found that the detection of object was also possible, up to distances of 1 m, in the anechoic chamber, while in the "conference" room detection could reach 2 m. On the other hand, in our study, 3 of the 5 depths were under 1 m, probably causing the absence of difference in results between the rooms. Moreover, we analyzed the ratio of the performance based on the learning, instead of considering the overall performance, like Schenkman and Nilsson did.

Interestingly, differences emerged between the two rooms in terms of accuracy and precision. The group of participants who completed the task in the reverberant room was both more accurate and more precise in their answers. A possible explanation for this result, in agreement with our hypothesis, is that the reverberant room is better at helping identify depths because the environment provides cues that are absent in the anechoic chamber.

To summarize, in both rooms depth can be learned in a similar way (learning gain for the reverberant room = 1.56 and for the anechoic room = 1.74; p = 0.98), but a difference in object depth estimation can be observed in terms of precision and accuracy. Why is this?

In an anechoic chamber most of the reflected sound waves come from the object of interest (early reflections) because the walls absorb any other residual sound. In a reverberant room, on the other hand, those residual waves are reflected, delayed or dampened by the walls. These waves generate a second set of echoes (late reflections), in the form of an exponentially decaying echo train. The ensemble of such echoes in principle is not the same when objects are placed at different depths. This suggests that some acoustical characteristics, such as late echoes and spectral coloration of the reverberant room, can be as important as the simpler, but more extensively studied, early echoes, which are known to be interpreted by our brain in terms of binaural cues [28, 29].

Based on our data, late reflections seem to play a crucial role in improving the precision and accuracy in a depth echolocation task. So our data support what Schenkman and Nilsson [5] call the "information-surplus principle", i.e. that often redundant information or information from many sources can give a more veridical perception [23], but this seems to hold true only when dealing with precision and accuracy of judgment. Moreover, the reverberation time of our reverberant room was much higher than the one used by Schenkman and Nilsson (respectively T_60_ = 1.4s and T_60_ = 0.4s). This suggests that, at least in the task used in this study, a room with a longer reverberation time does not impact negatively on performance, but rather improves it.

We would like to give a brief mention to the result of the closest depth (30cm) in Fig 5A for the anechoic chamber. Contrary to what would be expected, the error, for both precision and accuracy, at 30 cm is greater compared to that of further depths, while a positive correlation between increasing of error and increasing of depths would be expected, i.e. the further the distance, the greater the error. A possible explanation may be given by looking the results of the closest depth (30cm) in Fig 5B and the fact that the bar was located ahead of the participant’s face. Even if there is no global significant difference between finger-snap and mouth clicks, we found an interaction between the kind of click and position, at least in accuracy. Echolocation errors when finger-snapping are higher at 30cm as compared with the other depths. It might be that at very short distances the location of the sound source (e.g. the hand and not the mouth) interferes with an egocentric frame of reference not aligned with the hearing system. Specifically with the finger snap, the sound produced may not strike the center of the bar, which instead happens with the mouth clicks. Also, participants were free to move their hand which snapped. Our results show that moving a snapping hand may not be the best strategy when echolocating with close targets. The problem with snapping fingers at close distances does not seem to be a problem in the reverberant room: the richness of information provided by room reverberations (absent in the anechoic chamber) help to compensate for the comparatively poor information of the direct path, which is the only acoustic information to rely upon in the anechoic chamber.

The third result is that the type of clicking used by our sample (mouth click or finger-click) did not modulate our results: there was no significant difference between using finger-snaps or mouth-clicks while echolocating. From these results we can infer that probably the manner in which the sound is produced by novice echolocators does not matter in a depth task, but rather the amount of available environmental cues is the key factor (i.e. room reverberation). Probably the slight differences found in previous studies [25, 26], at an acoustical level, in which the mouth click seems to be the best sound for echolocation, followed by sound produced by the hand (finger snap or knuckle vacuum), might show up in tasks other than that used in our study. In any case, we emphasize that both techniques tested in this study are used effectively by expert echolocators.

To conclude, in the current study we have shown that even sighted individuals can learn to echolocate objects in depth: we found a significant improvement between the training and the second session. The amount of improvement was similar for the reverberant and the anechoic room. This supports the hypothesis that practice, rather than environmental cues, affects echolocation skills in novice sighted people. Even if we have demonstrated and confirmed with this work that training in an anechoic chamber allows participants to learn how to echolocate, the performance in terms of estimation accuracy and precision was higher in the reverberant room. Our study signifies advancement in the study of echolocation because it takes into consideration some aspects that have never before been considered in depth evaluation through echolocation.

**Fig 6:**
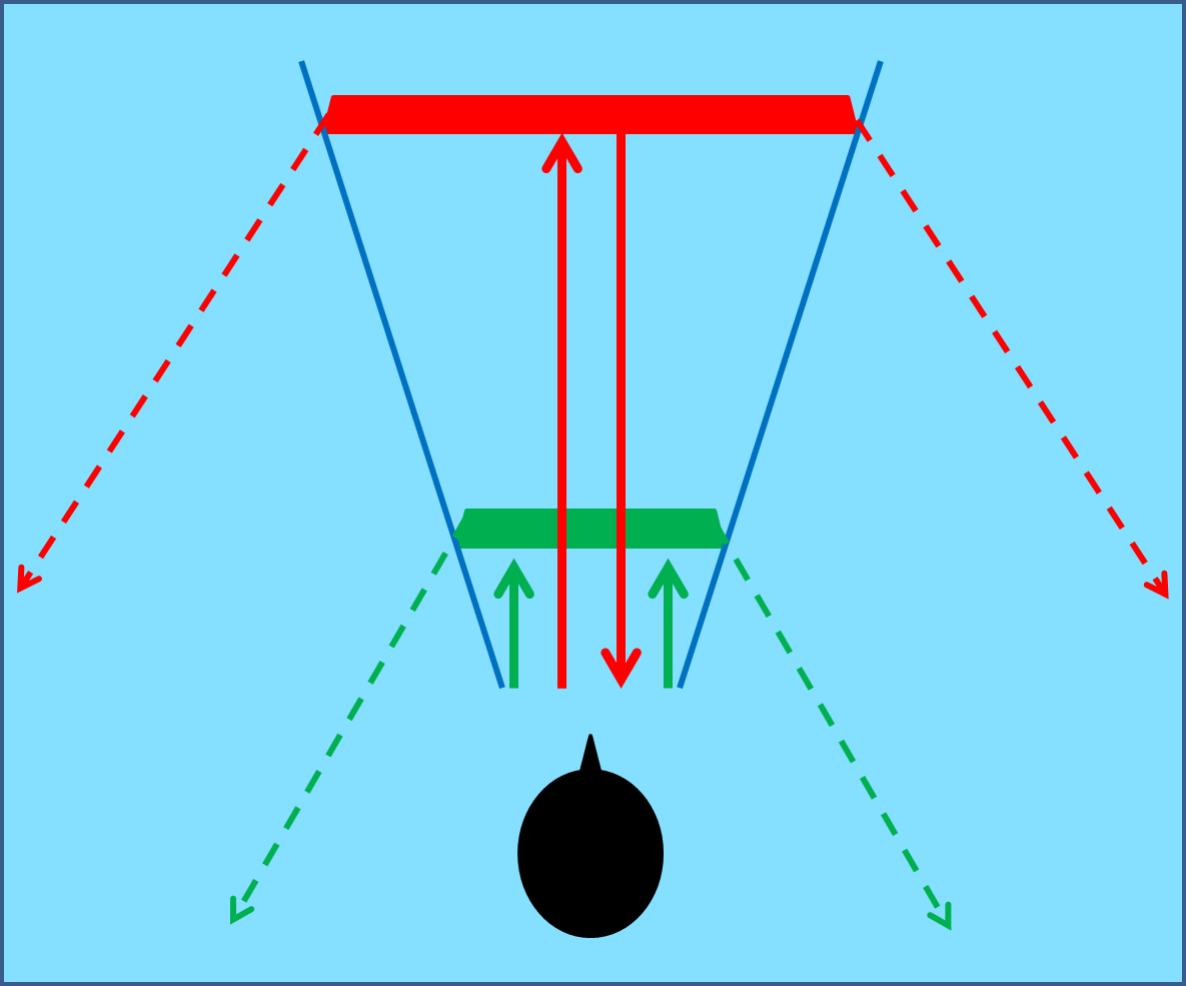
Qualitative explanation of the effect of size constancy on echoes. The subjects emit a click that is reflected by two targets, located at different distances but respecting size constancy. A constant acoustical angle (blue lines) ensures acoustical size constancy. However, the closer target generates a shorter early echo (green solid arrows) than the farther target (red solid arrows). Furthermore, the way late echoes spread in the room is greatly affected by the target distance: late echoes from the first target (green dashed arrows) only hit the wall behind the subjects, while those reflected by the farther target (red dashed arrows) collect the contribution of at least three walls.

Firstly, compared with Schenkman and Nilsson (2010), we chose the set of reflecting targets to match the same subtended angle: this was done to allow size to affect distance judgments only minimally, while maximizing the contribution of early echoes rather than late echoes. In fact, maintaining the same subtended angle ensures that the sound intensity multiplied by the target area (i.e. the sound power) is approximately constant. In other words, the “amount” of sound generated by the clicks that hits the target does not vary too much when changing the bars across trials in our setup [30]: what really changes is the relative delay between early reflections and late echoes in function of the target. This makes our study independent of the *energy* of the emitted sound, since the sound power over time (the sound energy) is approximately the same when hitting each one of our size-constant targets; however–and importantly–the distance at which targets are located greatly affects the way the reflected sound travels in the room over *space and time*. Both the early and the late echoes are greatly affected by distance. These are the acoustical features that this study aims to capture and that are likely to be at the core of echolocation skills. Fig 6 illustrates this qualitatively.

Secondly, compared with Schenkman and Nilsson [5], we also measured accuracy and precision together with the rate of correct responses, as they did. We considered a closer range of distances between the subject and the targets. We found that accuracy and precision improved after training but mainly in the closer position (30 cm), which Schenkman and Nilsson [5] did not investigate. A possible explanation for this result could be that echolocation, especially in closer positions, is not merely a matter of pulse-echo detection, but a perception of coloration of sound, because the sum of the emitted and the reflected sound determine how the sound is perceived. When this phenomenon is repeated, the listener may perceive a variation in the pitch, a phenomenon known as repetition pitch [31, 32], that is inversely proportional to the distance.

Thirdly, compared with Rowan et al (2013), we chose to place our targets in front of the subject to minimize the contribution of binaural cues in distance estimation tasks: both interaural level difference and interaural time difference are zero in our setup. In this way, early echoes may only contribute to distance estimation if the brain is actually able to estimate the delay between the moment the click is uttered and the moment the first echo arrives at both ears. However, we cannot deny that residual binaural cues may have influenced our task, since subjects were free to move their heads.

Many questions about how great the improvement in accuracy longitudinally remain unanswered, since we limited our study to three sessions. Although more studies are necessary to elucidate how echolocation can be used, the general evidence suggests that this technique can be learned and can bring great benefits to blind people [14, 17] as a reliable means to compensate for lack of vision. In this vein, this study suggests that learning echolocation in ecological environments such as real rooms may be the best way to proceed within rehabilitation practices. Although standards in teaching echolocation techniques do not exist yet, our results contribute to dispelling the myth that echolocation is an innate skill of blind individuals only. Admittedly, our study is circumscribed to depth estimation only. However, mapping depths with echoes is in principle a task not at all trivial for naïve sighted individuals. If naïve sighted subjects can learn the association between environmental sound echoes and object depths in just a few hours, then this learning may be extended to naïve blind individuals or to “de novo” blind persons who recently lost their sight.

## Supporting Information

S1 File Dataset (XLSX)
